# NSAIDs Overcome *PIK3CA* Mutation-Mediated Resistance to EGFR Inhibition in Head and Neck Cancer Preclinical Models

**DOI:** 10.3390/cancers14030506

**Published:** 2022-01-20

**Authors:** Hua Li, Noah D. Peyser, Yan Zeng, Patrick K. Ha, Daniel E. Johnson, Jennifer R. Grandis

**Affiliations:** Department of Otolaryngology—Head and Neck Surgery, University of California, San Francisco, CA 94143, USA; Hua.Li2@ucsf.edu (H.L.); Noah.Peyser@ucsf.edu (N.D.P.); Yan.Zeng@ucsf.edu (Y.Z.); patrick.ha@ucsf.edu (P.K.H.); daniel.johnson@ucsf.edu (D.E.J.)

**Keywords:** EGFR, NSAID, *PIK3CA*

## Abstract

**Simple Summary:**

Epidermal growth factor receptor (EGFR) inhibitors are approved by the Food and Drug Administration (FDA) for the treatment of several cancers including head and neck squamous cell carcinoma (HNSCC). It has been reported that activating mutations in *PIK3CA*, which are relatively common, may contribute to EGFR inhibitor resistance while conferring sensitivity to treatment with non-steroidal anti-inflammatory drugs (NSAIDs). We hypothesized that disruption of PI3K/COX/EGFR crosstalk via COX inhibition would overcome resistance to EGFR inhibition in HNSCC models, particularly in the context of *PIK3CA* mutation. We found that the combination of NSAIDs and EGFR inhibitors showed additive or synergistic effects in preclinical HNSCC models harboring mutant *PIK3CA*. Our results suggest that *PIK3CA* mutation in HNSCC might represent a predictive biomarker for EGFR inhibitors in combination with NSAIDs.

**Abstract:**

Epidermal growth factor receptor (EGFR) inhibitors are approved by the Food and Drug Administration (FDA) but remain under active clinical investigation for the treatment of both newly diagnosed and recurrent/metastatic head and neck squamous cell carcinoma (HNSCC). Despite EGFR expression in the majority of HNSCC tumors, the levels of total or phosphorylated EGFR have not consistently been correlated with a response to EGFR targeting agents. The lack of predictive biomarkers represents a major obstacle to successful use of these drugs. Activation of phosphatidylinositol 3-kinase (PI3K) signaling by mutation of the *PIK3CA* oncogene represents a plausible mechanism for EGFR inhibitor drug resistance. We compared the impact of EGFR inhibitors, alone or in combination with non-steroidal anti-inflammatory drugs (NSAIDs), in preclinical HNSCC models harboring mutant versus wild-type *PIK3CA*. Our results demonstrate additive or synergistic effects of NSAIDs and EGFR inhibitors in vitro and in vivo in *PIK3CA*-mutated HNSCC models. These findings suggest that the addition of NSAIDs to EGFR inhibitors for the treatment of HNSCC may represent a promising therapeutic strategy in *PIK3CA*-mutated cancers.

## 1. Introduction

Head and neck squamous cell carcinoma (HNSCC) is a heterogeneous and frequently fatal malignancy of the upper aerodigestive tract epithelium. Despite aggressive treatment, many patients develop therapeutic resistance leading to progressive disease, poor outcomes, and a dismal survival rate which has improved little in recent decades. HNSCC tumors express elevated levels of the epidermal growth factor receptor (EGFR), which has prompted the extensive clinical investigation of EGFR inhibitors, primarily monoclonal antibodies and small molecule tyrosine kinase inhibitors (TKIs). The Food and Drug Administration (FDA) approved cetuximab, a monoclonal antibody targeting the EGFR, for HNSCC patients in 2006, yet the clinical response to EGFR inhibition remains modest, despite the nearly ubiquitous EGFR overexpression in HNSCC tumors. EGFR TKIs are effective in selected patient populations. Clinical responses to EGFR inhibitors will likely be improved by the identification of predictive biomarkers and the development of well-tolerated co-targeting strategies.

The mutation or amplification of *PIK3CA*, which encodes the phosphatidylinositol 3-kinase alpha (PI3Kα) catalytic subunit, are among the most common genomic aberrations in HNSCC, with 37% (103 of 279) of cases exhibiting one or both alterations in The Cancer Genome Atlas (TCGA) report [1]. Our group and others have reported that activating mutations in *PIK3CA* may promote cetuximab resistance in HNSCC models [2,3]. Cetuximab resistance may also arise in HNSCC through alternative mechanisms, including via activation of other signaling pathways, such as the G-protein-coupled receptor (GPCR)-mediated autocrine release of EGFR ligands [4,5,6]. Evidence suggests that EGFR activation in turn leads to further activation of the phosphatidylinositol 3-kinase (PI3K) pathway and the continued secretion of the GPCR ligands, including prostaglandin E_2_ (PGE_2_), thereby promoting positive feedback and downstream oncogenic signaling [7]. PGE_2_ is the principle mediator of inflammation downstream of cyclooxygenase (COX) enzymes, which are markedly upregulated in HNSCC [8]. COX enzymes have been investigated thoroughly as targets for HNSCC prevention and treatment, using non-steroidal anti-inflammatory drugs (NSAIDs) alone, or in combination with other targeted therapies, including EGFR inhibitors [9,10,11]. The inhibition of EGFR [12] or COX [13] in HNSCC cells leads to increased PI3K pathway signaling via both GPCR-dependent and -independent mechanisms, suggesting PI3K activation may lead to a resistance to these monotherapies. Intriguingly, cumulative evidence has demonstrated a significant survival advantage in patients with *PIK3CA*-mutant, but not wild-type, colorectal cancer who began regular NSAID use (aspirin) following diagnosis [14]. Together, these data indicate intimate crosstalk between PI3K, COX, and EGFR, leading to robust oncogenic signaling.

We sought to test the hypothesis that the disruption of PI3K/COX/EGFR crosstalk via COX inhibition would overcome resistance to EGFR inhibition in HNSCC models, particularly in the context of *PIK3CA* mutation. We identified a panel of HNSCC cells with endogenous *PIK3CA* mutations and an intrinsic resistance to the EGFR TKIs erlotinib and lapatinib. We also engineered cells to overexpress *PIK3CA* constructs in order to validate our findings in an isogenic model system. Our results demonstrate that NSAID treatment markedly enhances sensitivity to EGFR TKIs in *PIK3CA*-mutant HNSCC cell line models, including isogenic models. We additionally report that the NSAIDs sulindac or indomethacin overcome EGFR TKI resistance in vivo using well-characterized patient-derived xenografts (PDXs), with more profound effects in *PIK3CA*-mutant models. Together, these results suggest that HNSCC patients may benefit from regular NSAID in combination with EGFR inhibitors, especially among patients with *PIK3CA*-mutant disease.

## 2. Results

### 2.1. PIK3CA-Mutant HNSCC Cells Are Resistant to EGFR Inhibition

**Figure 1:**
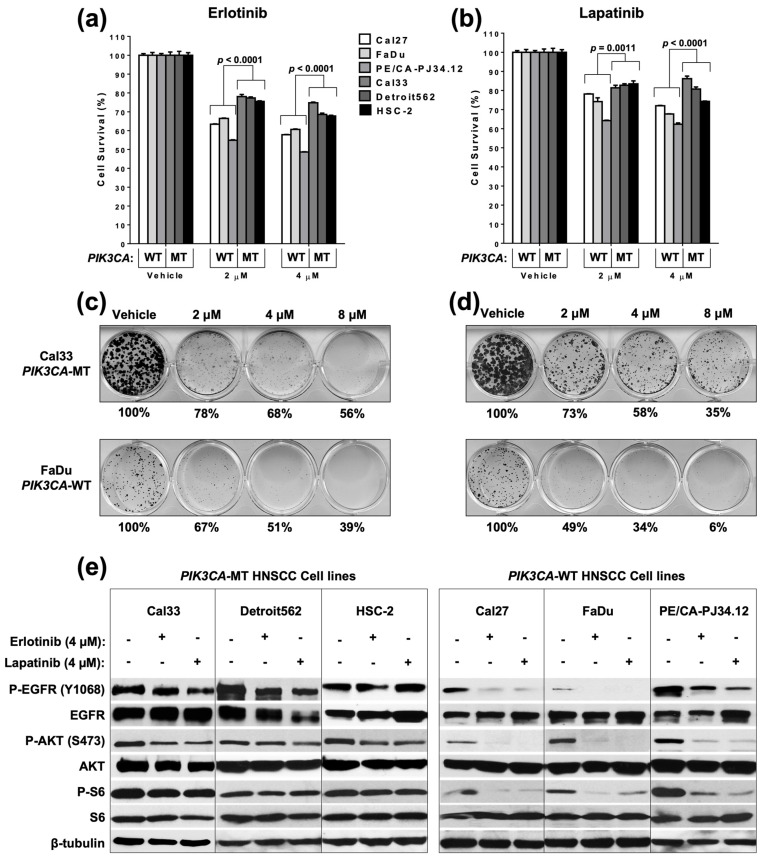
HNSCC cells with PIK3CA mutation are resistant to EGFR TKIs. HNSCC cell lines with WT PIK3CA (Cal27, FaDu, and PE/CA-PJ34.12) or PIK3CA mutation (p.H1047R; Cal33, Detroit562, and HSC-2) were treated with the indicated concentration of (**a**) erlotinib, or (**b**) lapatinib, for 48 h followed by MTT assay. *p* values were calculated using two-tailed *T*-tests with Welch’s correction, as appropriate. Bars represent mean + SEM. Cells were alternatively treated with the indicated concentrations of (**c**) erlotinib or (**d**) lapatinib for 10 days, followed by crystal violet staining. Percentages indicate the number of colonies relative to vehicle control counted using ImageJ software. (**e**) HNSCC cells were treated as indicated for 24 h followed by collection of whole cell lysate and immunoblot analysis.

As EGFR is a well-validated molecular target and upstream activator of PI3K in HNSCC, we first sought to test the contribution of PIK3CA mutation to sensitivity/resistance to EGFR TKIs in HNSCC cell line models. Treatment of a panel of PIK3CA wild-type [15] and PIK3CA-mutant (MT) (H1047R hotspot) cell lines with erlotinib (an EGFR-specific TKI; Figure 1a) or lapatinib (a dual EGFR/HER2 TKI; Figure 1b) indicated that PIK3CA-MT cells are resistant to EGFR inhibition, relative to PIK3CA-WT cells. Colony formation assays additionally indicated that PIK3CA-MT cells are resistant to erlotinib (Figure 1c) or lapatinib (Figure 1d), relative to PIK3CA-WT cells. Furthermore, while EGFR TKI treatment leads to only a modest reduction in EGFR/PI3K signaling in PIK3CA-MT HNSCC cells, p-EGFR (Y1068), p-AKT (S473), and p-S6 were markedly reduced in PIK3CA-WT cells following erlotinib or lapatinib treatment (Figure 1e). Together, these results indicate that PIK3CA mutation mediates resistance to EGFR inhibition via the sustained activation of EGFR/PI3K signaling in the presence of TKI in HNSCC cells. 

### 2.2. NSAIDs Overcome PIK3CA Mutation-Mediated Erlotinib Resistance

**Figure 2:**
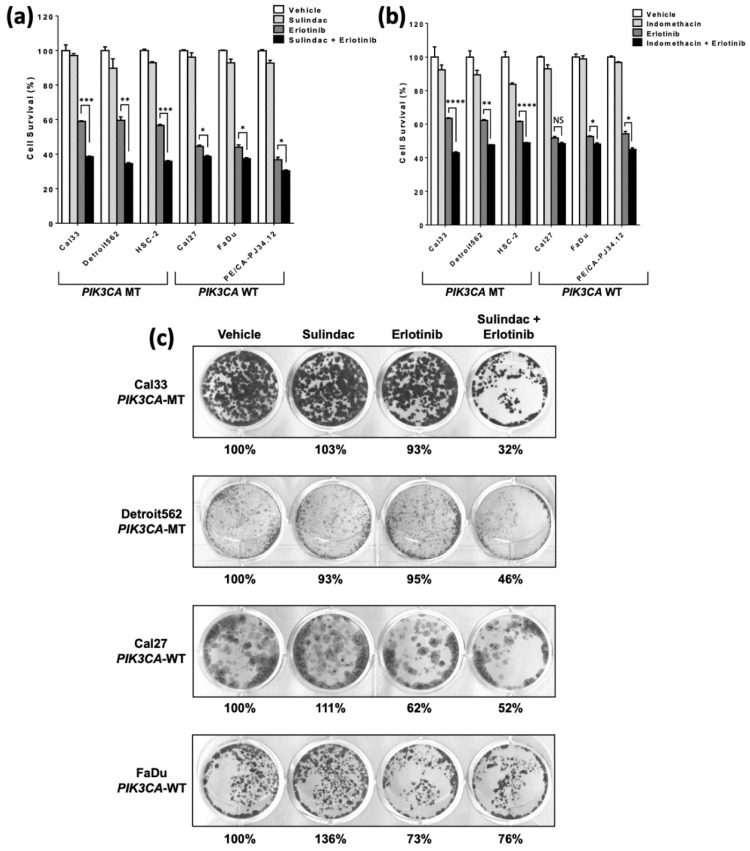
NSAID overcomes resistance to EGFR (erlotinib) inhibition. (**a**) HNSCC cell lines with WT PIK3CA (Cal27, FaDu, and PE/CA-PJ34.12) or PIK3CA mutation (p.H1047R; Cal33, Detroit562, and HSC-2) were treated with vehicle, sulindac (100 μM), erlotinib (2 μM), or a combination of sulindac and erlotinib for 48 h, followed by MTT assay. (**b**) Cells were treated as in panel A with substitution of 100 μM indomethacin for sulindac. *p* values were calculated using a two-tailed *T*-test with Welch’s correction. * *p* < 0.05; ** *p* < 0.005; *** *p* < 0.0005; **** *p* < 0.0001; NS: not significant. Bars represent mean + SEM. (**c**) Longer-term survival was assessed by seeding an equal number of cells in 12-well plates followed by treatment, as indicated. After 10 days, cells were visualized by crystal violet staining and counted using ImageJ software. Percentages indicate the number of colonies relative to vehicle control.

Since PIK3CA mutation is associated with resistance to EGFR TKIs as well as sensitivity to NSAIDs [16], we next sought to test the hypothesis that NSAIDs would overcome erlotinib resistance in PIK3CA-mutant HNSCC cells. We treated our panel of WT or MT PIK3CA cell lines with vehicle, NSAID (sulindac or indomethacin), erlotinib, or a combination. Consistent with our previous results, PIK3CA-MT cell lines were resistant to erlotinib alone, relative to PIK3CA-WT cells (Figure 2a,b; *p* < 0.0001 for both drugs, two tailed *T*-tests). Sulindac or indomethacin alone had modest effects on cell survival at the low concentrations tested, yet still significantly enhanced sensitivity to erlotinib in both PIK3CA-WT and -MT cell lines, as determined by MTT assays (Figure 2a,b). In all cases, PIK3CA-MT cells were more sensitive to NSAID treatment. Notably, while PIK3CA-mutant cells were relatively resistant to erlotinib alone, the combination with NSAID led to similar levels of cell survival in both PIK3CA-MT and -WT cells, indicating that the addition of NSAIDs effectively overcame the PIK3CA-mediated erlotinib resistance in PIK3CA-MT cells.

**Figure 3:**
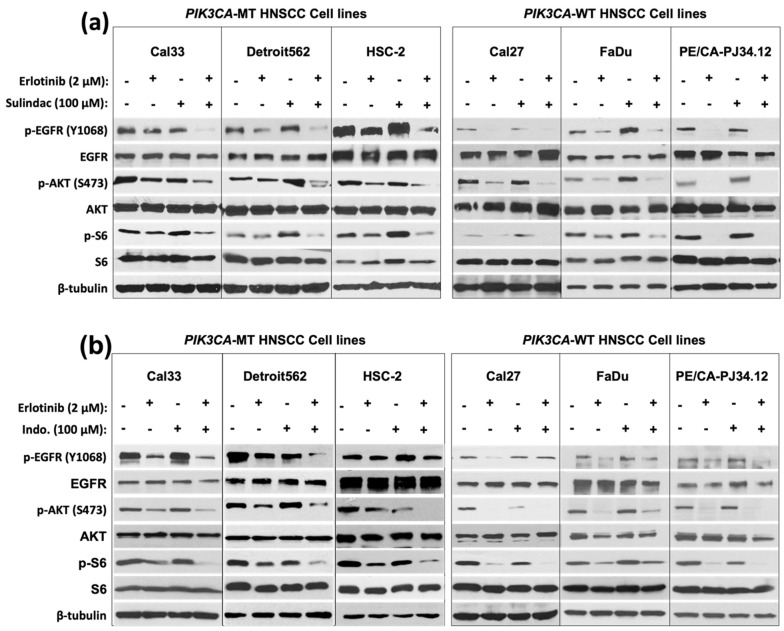
Combination of NSAIDs with EGFR TKI abrogates downstream signaling. HNSCC cell lines with WT PIK3CA (Cal27, FaDu, and PE/CA-PJ34.12) or PIK3CA mutation (H1047R; Cal33, Detroit562, and HSC-2) were treated with vehicle, sulindac (100 μM), indomethacin (100 μM), erlotinib (2 μM), or a combination of sulindac and erlotinib (**a**), or indomethacin and erlotinib (**b**), for 24 h followed by collection of whole cell lysate and immunoblot analysis. The uncropped Western blots have been shown in Figure S2.

We also observed NSAID and erlotinib synergy in colony formation assays following 10 days of treatment, where PIK3CA-MT cells were more inhibited by the combination than PIK3CA-WT cells were (Figure 2c). Next, we assessed EGFR/PI3K signaling in our cell line panel following vehicle, erlotinib, NSAID, or combination treatment, and found that erlotinib alone was sufficient to abrogate pathway signaling in PIK3CA-WT, but not -MT, cell lines (Figure 3a,b). The addition of NSAID led to only modest further reduction of EGFR/PI3K signaling in PIK3CA-WT cells. In contrast, the combination led to a robust downregulation of p-EGFR (Y1068), p-AKT (S473), and p-S6 in PIK3CA-MT cells. Together, these results demonstrate that NSAIDs enhance the response to EGFR TKI and abrogate PIK3CA mutation-mediated resistance to EGFR inhibition in HNSCC cells, by the downregulation of EGFR/PI3K signaling.

**Figure 4:**
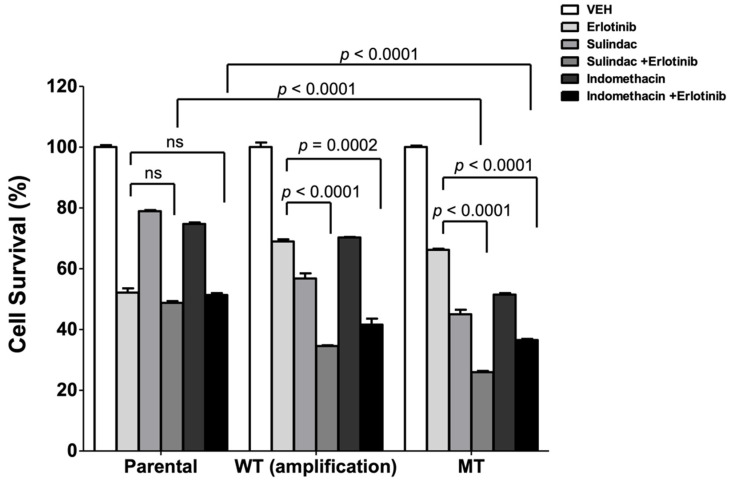
Combination of NSAID with erlotinib markedly inhibits cell survival in isogenic HNSCC cells overexpressing mutant PIK3CA. PCI-52 parental cells and isogenic cells overexpressing WT or MT (H1047R) PIK3CA were treated with vehicle, erlotinib (2 μM), sulindac (100 μM), indomethacin (100 μM), or combination of sulindac and erlotinib or indomethacin and erlotinib for 48 h followed by MTT assay. *p* values were calculated using a two-tailed *T*-test. Bars represent mean + SEM. ns: not significant.

To further validate these findings, we engineered PCI-52 cells to overexpress WT or MT PIK3CA and treated them with vehicle, erlotinib, NSAID, or a combination. This cell line was selected because it contains wild-type, unamplified PIK3CA. As anticipated, cells overexpressing MT PIK3CA (H1047R) or WT (analogous to gene amplification) were both resistant to erlotinib and sensitive to sulindac or indomethacin, relative to parental cells (Figure 4). Furthermore, while the parental cells did not respond to the combination more robustly than erlotinib alone, cells overexpressing WT or MT PIK3CA were significantly more responsive to the combination of erlotinib with NSAID. Moreover, the combined treatment led to significantly lower cell survival in both WT and MT PIK3CA-overexpressing cells relative to parental cells, although no substantial differences between WT and MT were detected on immunoblots for p-EGFR and pAKT (Figure S1). These findings confirm, in an isogenic model, that PIK3CA mutation (and likely amplification) is associated with erlotinib resistance, NSAID sensitivity, and a reversal of erlotinib resistance upon combination treatment.

**Figure 5:**
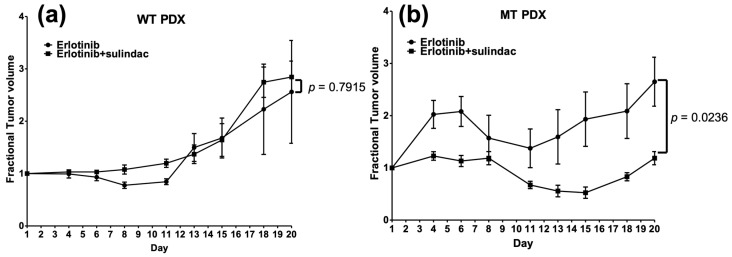
A combination of sulindac with erlotinib potently inhibits tumor growth in *PIK3CA*-mutant PDX. PDXs were implanted into the flanks of NSG mice and allowed to grow until palpable (n = 4 tumors per group). Mice were then treated with erlotinib (50 mg/kg by oral gavage every day), or the combination of sulindac (30 mg/kg by IP injection every other day) and erlotinib. (**a**) A *PIK3CA*-WT PDX model does not exhibit significant response to the combination relative to erlotinib alone. (**b**) A *PIK3CA*-MT PDX model exhibits significant growth inhibition upon the combination of sulindac with erlotinib treatment relative to erlotinib alone. *p* values were calculated on the last day using two-tailed *T*-tests with Welch’s correction, as appropriate. Data are displayed as mean ± SEM.

Lastly, we sought to test the combination in a more clinically relevant model. We therefore established one PIK3CA-MT (p.E542K) and one PIK3CA-WT PDX in NSG mice and treated with vehicle, sulindac, erlotinib, or the combination as described in the Methods section (Figure 5). We previously reported the lack of response in these PDX models to vehicle control treatment [16]. Similar to our in vitro studies, sulindac led to significant tumor growth inhibition only in the PIK3CA-MT PDX model. Furthermore, the combination of erlotinib and sulindac led to synergistic growth inhibition in PIK3CA-MT, but not PIK3CA-WT, PDX models. Together, these results indicate that PIK3CA mutation may predict exquisite sensitivity to the combination of EGFR inhibition with NSAID in HNSCC. 

## 3. Discussion

EGFR is a proven therapeutic target in HNSCC, due to overexpression in patient tumors and the demonstrated clinical activity of EGFR inhibitors [17]. Agents that target EGFR are generally less toxic compared with conventional chemotherapy and patients who cannot tolerate platinum-based chemotherapy can be treated with the EGFR monoclonal antibody cetuximab. However, the activity of EGFR inhibitors alone is relatively modest and limited to a minority of patients, and in the absence of a reliable predictive biomarker of response, it is unclear how to identify HNSCC patients most likely to benefit from EGFR-targeted agents [18,19]. 

There is a paucity of clinical trial data to support the routine use of EGFR tyrosine kinase inhibitors (TKIs) for the prevention or treatment of HNSCC. In the absence of a validated biomarker of response or resistance to EGFR TKI, all therapeutic studies carried out to date have administered these agents, alone or in combination, to unselected patient populations with no indication of robust activity [20]. We previously reported a patient with an exceptional response to erlotinib monotherapy on a window-of-opportunity trial. Sequencing of the tumor combined with preclinical modeling studies suggested that an uncommon MAPK1 mutation found in 1–2% of HNSCC patients (E322K) mediated this clinical response [21]. In the absence of activity in unselected HNSCC populations, there is little justification to administer EGFR TKI for the treatment of this cancer.

Erlotinib has also been studied in combination with celecoxib and radiation in recurrent HNSCC with demonstration of clinical activity and feasibility [22]. However, in the absence of additional data comparing this regimen with standard of care, the future of the combination is unknown. We previously reported the impact of celecoxib, alone or in combination with sulindac, in a window-of-opportunity trial in HNSCC patients undergoing surgical resection with curative intent [11]. While the brief course of therapy prior to surgery was well tolerated, we saw no impact of the addition of the NSAID to erlotinib therapy on the inhibition of proliferation in the tumor. In the modern era of cancer immunotherapy, the impact of EGFR TKI plus NSAIDs in the setting of anti-PD-1/PDL-1 is likely needed to impact clinical practice.

EGFR TKI have also been investigated in the setting of HNSCC chemoprevention. Saba et al. carried out a phase 1b trial testing the impact of erlotinib and celecoxib in patients with advanced premalignant lesions [10]. Although there were encouraging responses with the combination therapy, erlotinib-associated rash proved to be a dose-limiting toxicity. 

Alterations in the *PIK3CA* pathway are common in HNSCC, and studies have linked *PIK3CA* mutations to poorer survival in patients with head and neck cancer [23], as well as other solid tumors [24]. Moreover, in HPV-positive patients, those with *PIK3CA* mutations were recently found to have an increased chance of recurrence, and worse disease-free survival rates [25]. Preclinical studies have indicated that the dual inhibition of the *PIK3CA* and EGFR pathways demonstrates synergistic cell killing in head, neck [26] and lung cancer models [27]. 

*PIK3CA*-activating mutations have also been studied in colorectal cancer (CRC), where they are commonly identified in 20% of patients [28]. In patients with CRC, the use of aspirin has been consistently shown to reduce the risk of cancer development [29], as well as cancer-specific mortality [30]. Further studies determined that this protective effect of NSAID use was closely associated with *PIK3CA* mutation in patient tumors [14,31]. 

A similar benefit of NSAID use in HNSCC has been postulated, but the results of observational studies remain somewhat mixed [32,33,34], and findings were not stratified according to mutational profiles. We recently reported a survival benefit in HNSCC patients with *PIK3CA* alterations who used NSAIDs on a regular basis [16]. On multivariate analysis, the survival benefit of NSAID use was statistically significant, but only in patients whose tumors harbored *PIK3CA* mutation or amplification. Activation of the PI3K pathway could represent both a therapeutic target, as well as a mechanism of resistance to certain classes of drugs. In HNSCC in vitro and PDX models, mutant *PIK3CA* led to increased sensitivity to NSAID treatment, mediated at least in part, through prostaglandin E2 production [16]. 

Additional candidate predictive biomarkers that could be explored include cancer stem cells. One group examined the effects of cetuximab and erlotinib on stem cell sub-populations in HNSCC cell lines, and found that these EGFR inhibitors induced large shifts of cells between the epithelial and mesenchymal cancer stem cell populations [35]. Others have reported that the EGFR/HER2 TKI afatinib sensitizes HNSCC cells to radiation by specifically targeting cancer stem cells [36]. One plausible mechanism for these effects was demonstrated by a study by Seguin et al., showing that a complex containing integrin α(v)β_3_, KRAS and RalB mediated tumor stemness in conjunction with resistance to EGFR TKI in carcinomas of the breast, lung and pancreas [37]. The impact of EGFR TKI, alone or in combination with NSAIDs in the setting of PIK3CA alterations remains largely unexplored. Finally, platinum remains a standard of care chemotherapeutic agent for HNSCC treatment. Studies to date, combining EGFR TKI and platinum, have shown the benefits to overall survival of patients with HNSCC [38]. The role of NSAIDs in combination with EGFR TKI and platinum remains untested.

In the present study, we demonstrated that endogenous *PIK3CA*-MT HNSCC cell lines show an enhanced sensitivity to combined EGFR–NSAID treatment relative to *PIK3CA*-WT cell lines. The combination also led to decreased EGFR/PI3K pathway activation, specifically in *PIK3CA*-MT cell lines. Engineered isogenic mutant *PIK3CA* PCI-52 cells also showed the same pattern of sensitivity to combined EGFR–NSAID treatment, compared with wild-type cells. Ultimately, these findings were confirmed in an in vivo PDX model treated with erlotinib and sulindac, where there was increased growth inhibition primarily in the *PIK3CA*-MT tumors.

EGFR TKI were granted orphan drug status by the European Medicines Agency for the treatment of HNSCC in the setting of Fanconi Anemia based on promising findings in cell line xenograft studies [39]. The vast majority of HNSCCs arise in individuals without Fanconi Anemia, and the role of EGFR and NSAIDs remains unproven. The precision medicine is anchored by the probability that patient and tumor characteristics can be leveraged to guide treatment. Our results suggest that PIK3CA mutation in HNSCC may serve as candidate for predictive biomarkers. A prospective evaluation of HNSCC patients is needed to directly test this possibility.

## 4. Materials and Methods

### 4.1. Cell Lines

HNSCC cell lines harboring endogenous WT or MT *PIK3CA* were identified using the Cancer Cell Line Encyclopedia [40]. Cal27, FaDu, and Detroit562 were obtained from ATCC (Manassas, VA, USA). PE/CA-PJ34.12 (clone 12) were obtained from Sigma-Aldrich (St. Louis, MO, USA). HSC-2 were obtained from Dr. Hideo Niwa (Nihon University, Tokyo, Japan). Cal33 cells were obtained from Dr. Gerard Milano (Nice Cancer Center, Nice, France). PCI-52 cells were obtained from Dr. Theresa Whiteside at the University of Pittsburgh (Pittsburgh, PA, USA). All cell lines were verified by short tandem repeat profiling performed by Genetica DNA Laboratories (Cincinnati, OH, USA). Cal27, FaDu, Detroit562, HSC-2, Cal33, and PCI-52 cells were maintained in DMEM (Corning, Corning, NY, USA) with 10% FBS (Gemini Bio-Products, West Sacramento, CA, USA). PE/CA-PJ34.12 were maintained in Iscove’s DMEM (Corning, Corning, NY, USA) with 10% FBS and 2 mM L-glutamine (Life Technologies, Carlsbad, CA, USA). All cells were cultured in a humidified incubator at 37 °C and 5% CO_2_.

### 4.2. Stable Cell Line Generation

*PIK3CA* plasmids were obtained from Dr. Gordon Mills (University of Texas MD Anderson Cancer Center, Houston, TX, USA) and were used to subclone *PIK3CA* into the doxycycline-inducibe pLVX-Puro (Clontech, Mountain View, CA, USA). Each vector generated was verified by Sanger sequencing (Quintara Biosciences, South San Francisco, CA, USA). The Lenti-X Tet-One Inducible expression system was used to generate lentivirus according to the manufacturer’s instructions (Clontech). After infection with lentivirus, PCI-52 cells were subject to puromycin selection (0.5 µg/mL) for a period of weeks, and PI3K overexpression was verified by immunoblot following 48 h of incubation in doxycycline (1 µg/mL).

### 4.3. Drug Treatment

Cells were plated at 10,000 cells per well in 24-well plates and allowed to adhere overnight. The following day cells were treated in triplicate with vehicle (DMSO), erlotinib (Tarceva, Astellas Pharm Global Development Inc. Northbrook, IL USA), lapatinib (Sigma-Aldrich, St. Louis, MO, USA), sulindac sulfide (Sigma-Aldrich, St. Louis, MO, USA), or indomethacin (Sigma-Aldrich, St. Louis, MO, USA) at the indicated concentration of drug for 48 h. Cell growth inhibition was determined by MTT assay and normalized to vehicle-treated wells.

### 4.4. Apoptosis Assays

Apoptosis was analyzed by counting cells with condensed chromatin and micro-nucleation following nuclear staining with Hoechst 33258 (Invitrogen, Waltham, MA, USA). A minimum of 300 cells were analyzed for each sample. Fluorescence images were captured with the EVOS FL microscope (Life Technologies, Carlsbad, CA, USA).

### 4.5. Colony Formation Assays

*PIK3CA*-MT (Cal33) or -WT (FaDu) cells were plated at 1000 cells per well in 12-well plates. Cells were treated with vehicle control, or the indicated drug or a combination. After 10 days, colonies were visualized by crystal violet staining and counted using ImageJ software (ImageJ1, NIH, Bethesda, MD, USA).

### 4.6. Immunoblotting

Primary antibodies targeting p-AKT (S473), AKT, p-EGFR (Y1068), EGFR, p-S6, and S6 were purchased from Cell Signaling Technology (Boston, MA, USA). β-tubulin primary antibody was purchased from Abcam (Cambridge, MA, USA). Secondary antibodies were purchased from Bio-Rad (Hercules, CA, USA). 

### 4.7. Animals

All animal procedures were performed in accordance with protocols approved by Institutional Animal Care and Use Committee of the University of California, San Francisco (IACUC protocol: AN187611) or the University of Pittsburgh (IACUC protocol: 13072053). Additionally, 5–6-week-old NOD.Cg-*Prkdc*^scid^ *Il2rg^tm1Wjl^*/SzJ (NSG) mice were obtained from Jackson Laboratories (Bar Harbor, ME) or the UCSF Breeding Core Facility. Patient-derived xenografts (PDXs) were implanted into one flank of NSG mice under anesthesia and allowed to grow until palpable (~100 mm^3^). Mice (four mice with four tumors for each treatment group) were treated with vehicle control (saline), sulindac sulfide intraperitoneally (30 mg/kg; Sigma-Aldrich, St. Louis, MO, USA), erlotinib by oral gavage (50 mg/kg), or the combination of both as indicated throughout. Tumors were measured every other day by Vernier caliper in two dimensions and the volume was calculated as (larger measurement) × (smaller measurement)^2^/2.

### 4.8. Statistics

Two-tailed *T*-tests were used for normally distributed data (determined by D’Agostino–Pearson omnibus normality test) with equivalent between-group variances. Welch’s correction was used to correct for unequal between-group variance as appropriate. Two-tailed Mann–Whitney tests were used when data were non-Gaussian.

## 5. Conclusions

These findings provide further mechanistic evidence that *PIK3CA* alterations could be used to successfully predict the response to EGFR inhibition in combination with NSAIDs. By effectively using *PIK3CA* mutation/amplification as a biomarker for EGFR inhibitor response, one could envision using NSAIDs in combination with EGFR inhibitors to provide clinical benefit to a significant portion of patients, while adding little to their side effect burden. The data presented here support the use of NSAIDs in patients receiving EGFR inhibitor therapy in HNSCC, particularly those whose tumors harbor alterations in *PIK3CA* or the PI3K signaling pathway. 

## Data Availability

Data is contained within the article or supplementary material. The data presented in this study are available in this article.

## Supplementary Materials

The following are available online at https://www.mdpi.com/article/10.3390/cancers14030506/s1, Figure S1: Treatments of EGFR TKI and NSAIDs changes the pathway signaling in engineered HNSCC PCI-52 cells, Figure S2: The uncropped Western blots.
