# Supplementary material for: NSAIDs Overcome PIK3CA Mutation-Mediated Resistance to EGFR Inhibition in Head and Neck Cancer Preclinical Models
Source: Cancers (Basel). 2022 Jan 20;14(3):506. doi: 10.3390/cancers14030506 (PMC8833811; doi:10.3390/cancers14030506)
Supplement: Supplementary file 1 [file cancers-14-00506-s001.zip › cancers-1495009-supplementary.pdf]

cancers MDPI

Supplementary Materials: NSAIDs Overcome *PIK3CA* Mutation-Mediated Resistance to EGFR Inhibition in Head and Neck Cancer Preclinical Models

Hua Li, Noah D. Peyser, Yan Zeng, Patrick K. Ha, Daniel E. Johnson and Jennifer R. Grandis

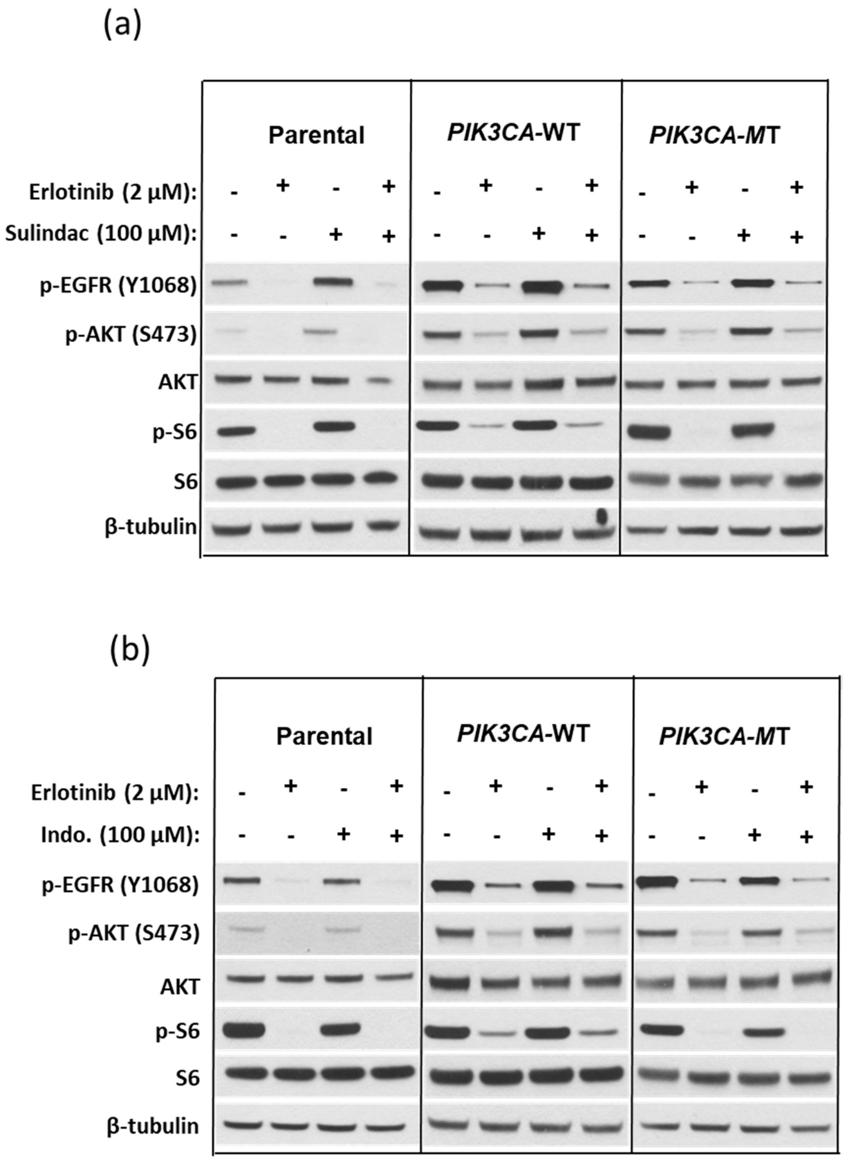

Figure S1. Treatments of NEGFR TKI and NSAIDs changes the pathway signaling in engineered HNSCC PCI-52 cells. The HNSCC cell line PCI-52 parental cells, PCI-52 cells with overexpressed WT PIK3CA or PIK3CA mutation (H1047R) were treated with vehicle, sulindac (100 µM), indomethacin (100 µM), erlotinib (2 µM), or combination of sulindac and erlotinib (a) or indomethacin and erlotinib (b) for 24 hours followed by collection of whole cell lysate and immunoblot analysis.

(A)

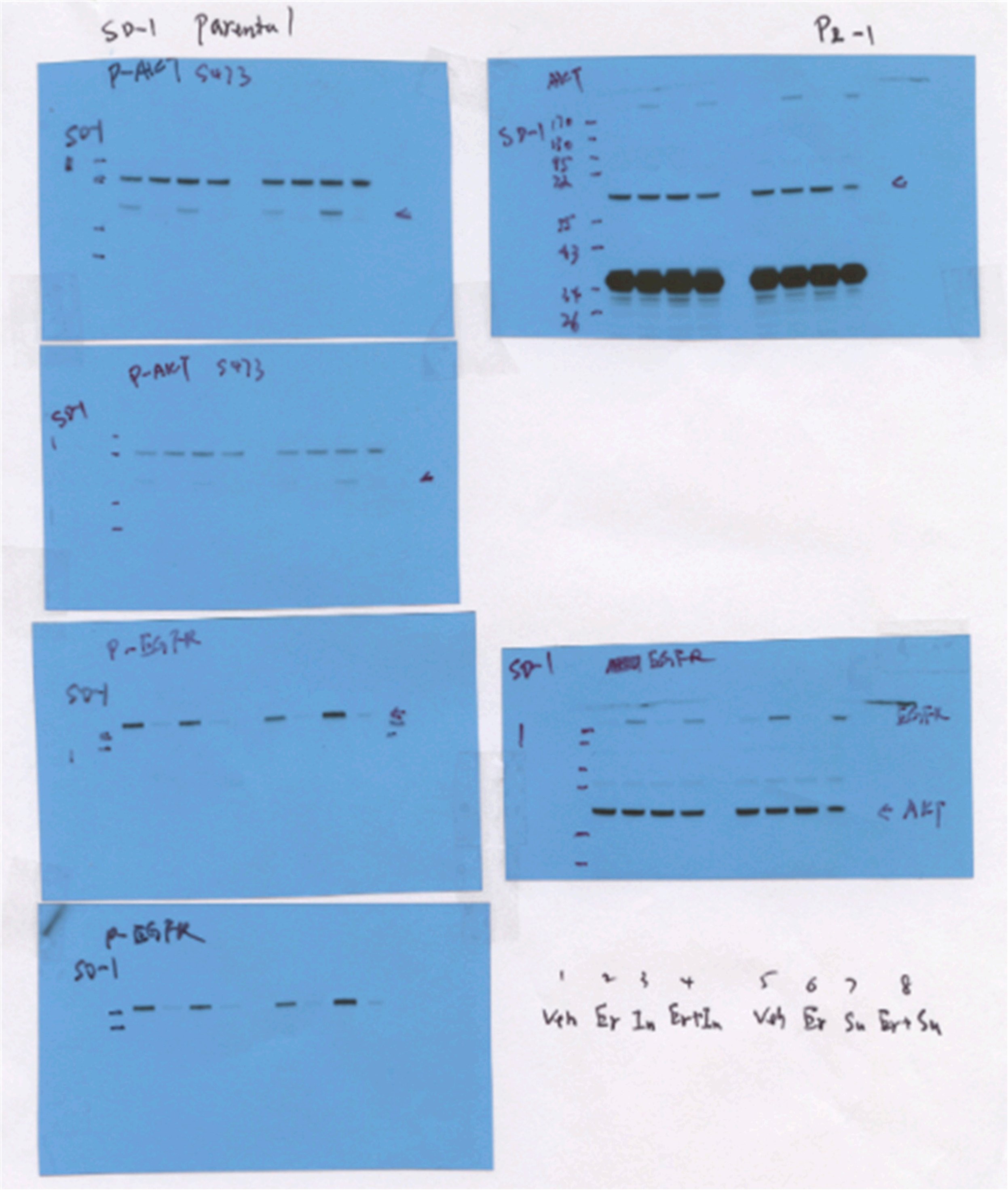
SD-1 Parental
P-AKT S473
AKT
SD-1
170
130
95
72
55
43
34
26
P-AKT S473
P-EGFR
SD-1
EGFR
AKT
P-EGFR
SD-1
1 2 3 4 5 6 7 8
Veh Er In Er+In Veh Er Su Er+Su

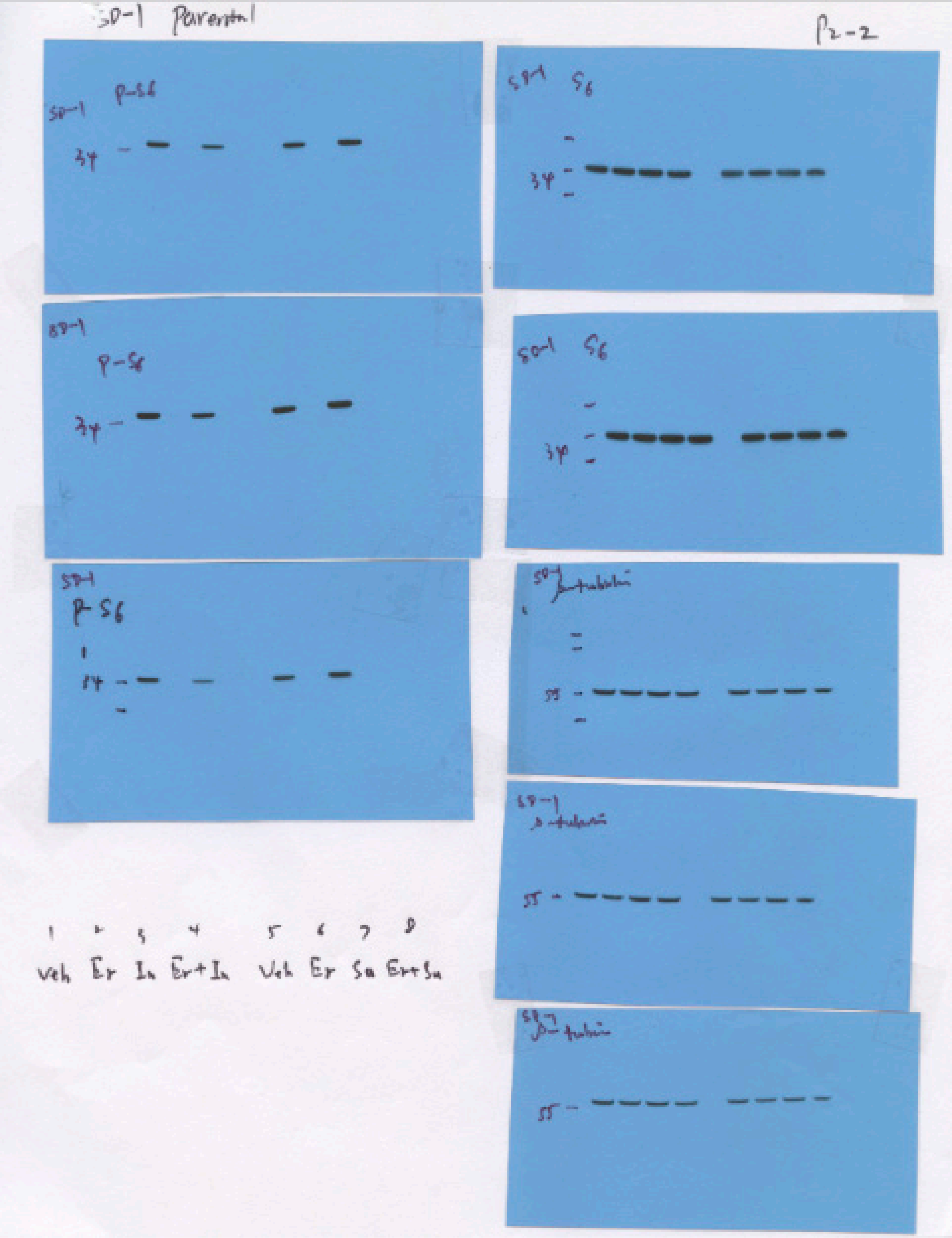
SD-1 Parental
P2-2
SD-1 P-S6
34
SD-1 S6
34
SD-1 P-S6
34
SD-1 S6
34
SD-1 P-S6
34
SD-1 β-tubulin
55
SD-1 β-tubulin
55
SD-1 β-tubulin
55
1 2 3 4 5 6 7 8
Veh Er In Er+In Veh Er Su Er+Su

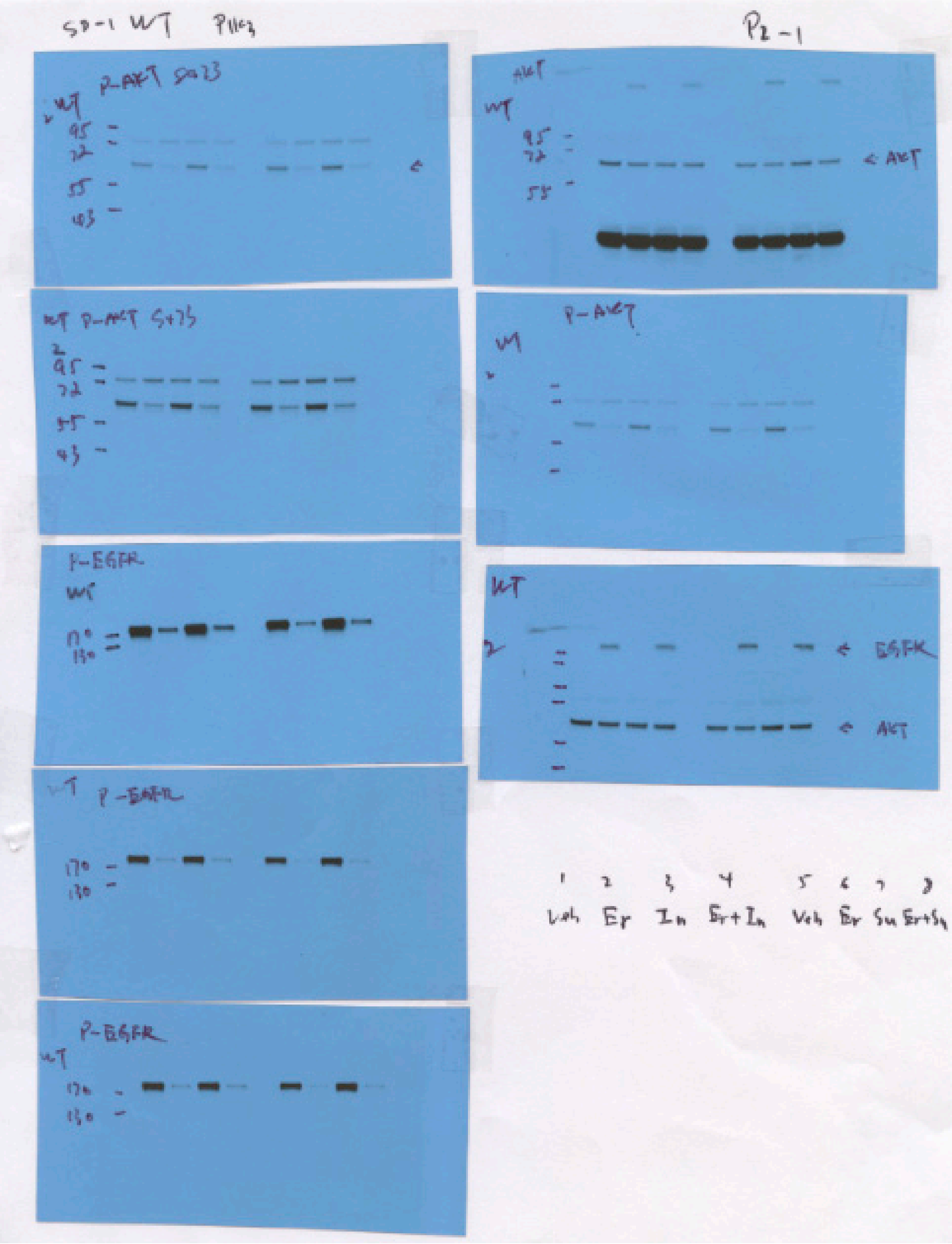
P2-1
P-AKT
WT
95
72
55
43
AKT
P-AKT S473
P-EGFR
170
130
EGFR
1 2 3 4 5 6 7 8
Veh Er In Er+In Veh Er Su Er+Su

SD-1 WT PIk3 P2-2

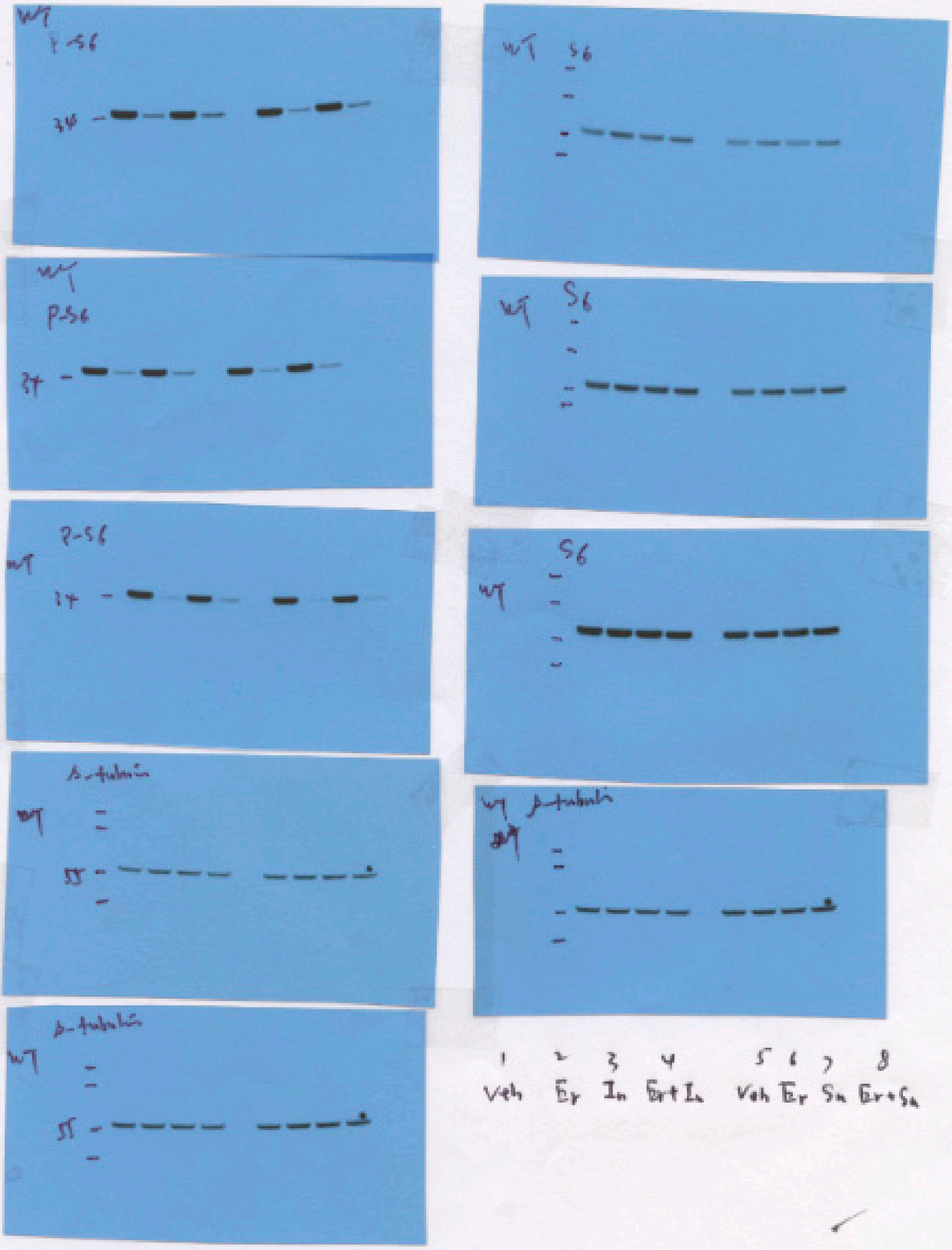

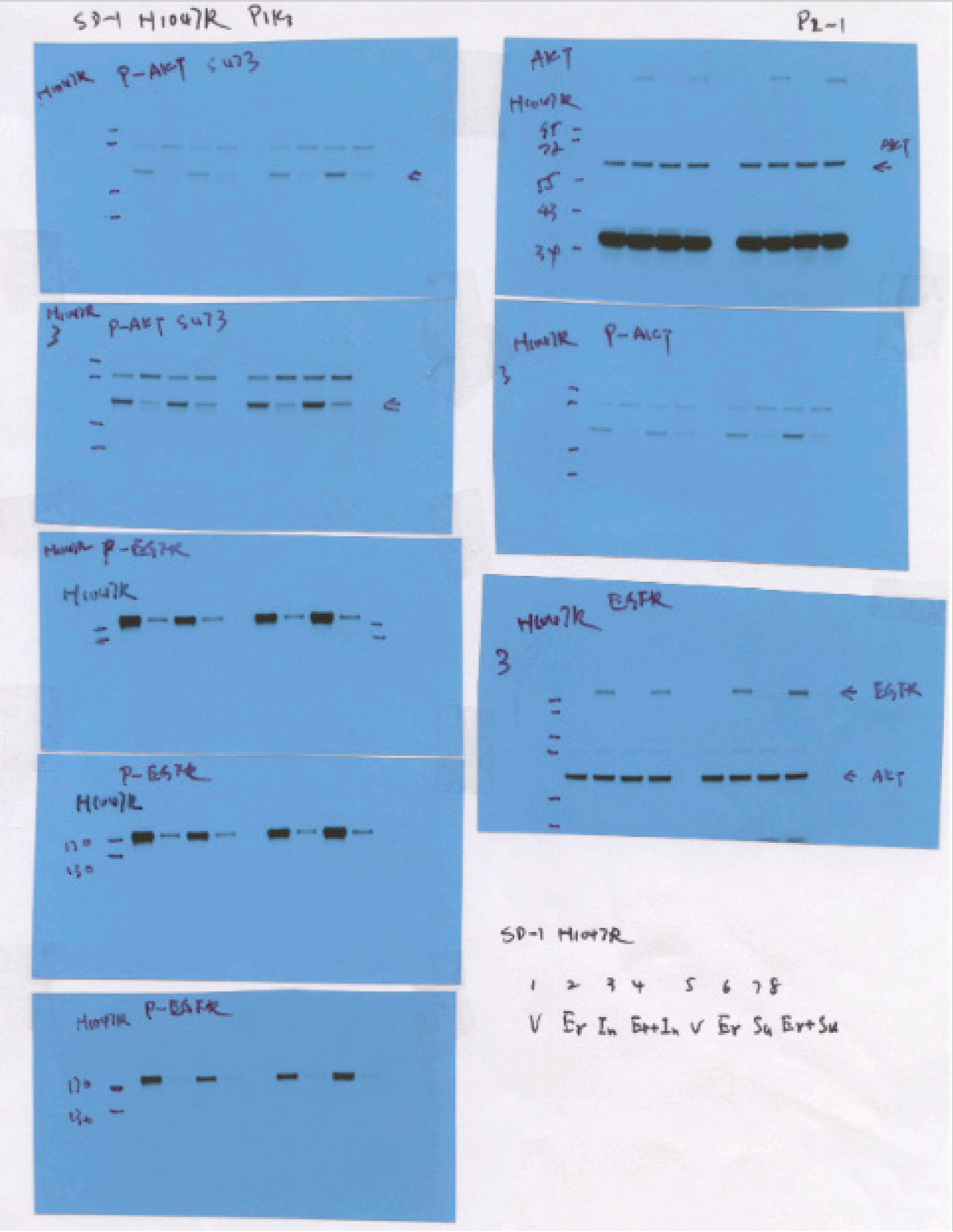
SD-1 H1047R PIK3
P2-1
H1047R P-AKT S473
AKT
H1047R
95
72
55
43
34
AKT
H1047R
3
P-AKT S473
H1047R P-AKT
3
H1047R P-EGFR
H1047R
H1047R EGFR
3
EGFR
AKT
P-EGFR
H1047R
170
130
H1047R P-EGFR
170
130
SD-1 H1047R
1 2 3 4 5 6 7 8
V Er In Er+In V Er Su Er+Su

SD-1 H1047R PIK3 P2-2

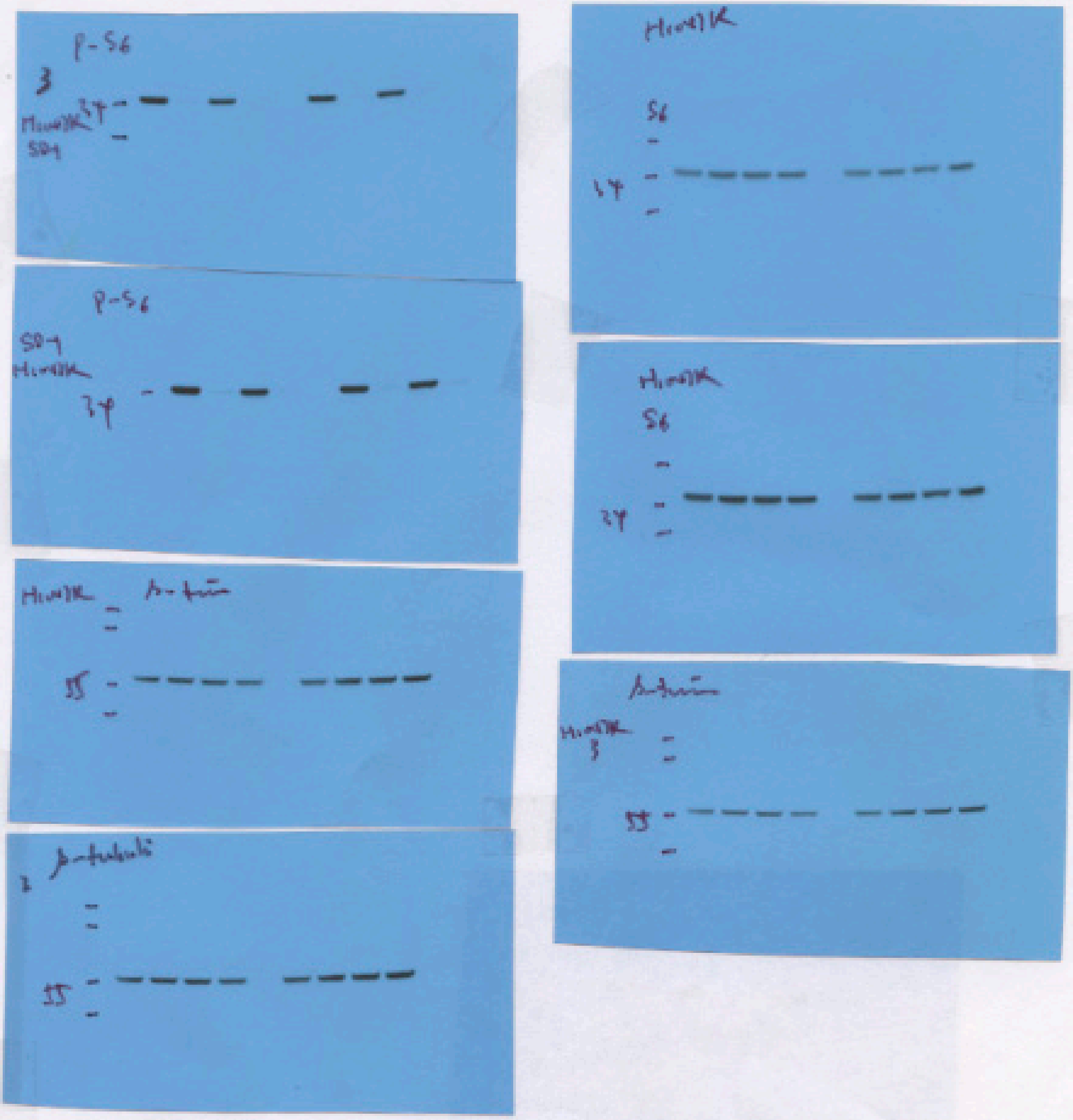

SD-1 H1047R ~~[illegible]~~. PIK3

1 2 3 4 5 6 7 8

Veh Er Su Er+Su V Er Su Er+Su

(B)

Fig3. B

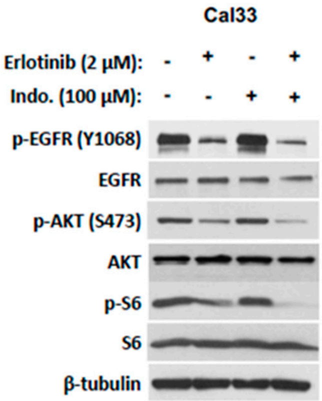

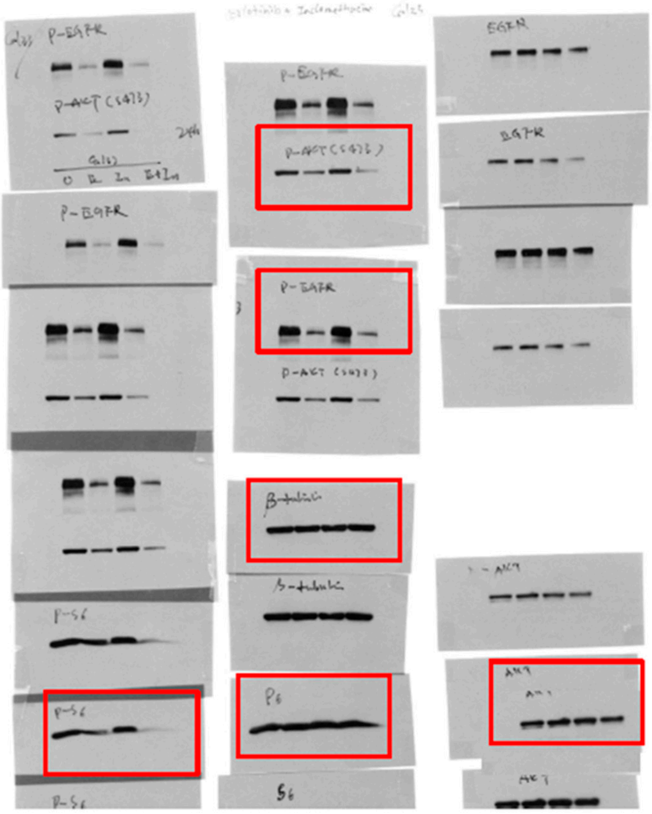

The red squares in the right image show the original ones in the left figures

Fig3. B

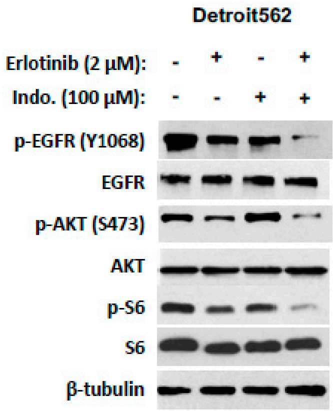

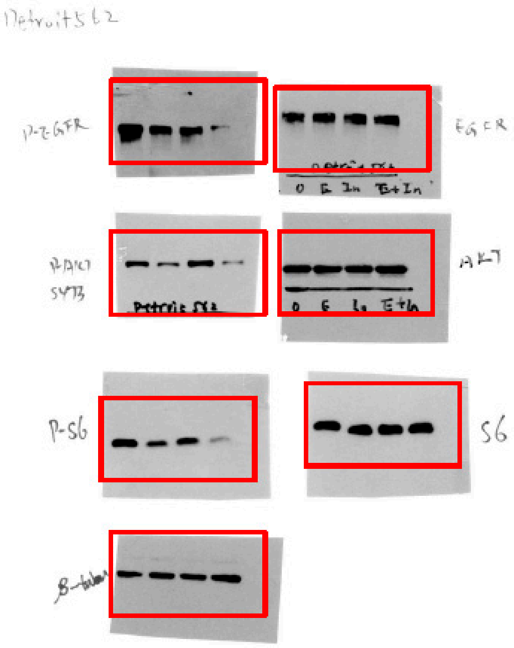

The red squares in the right image show the original ones in the left figures

Fig3. B

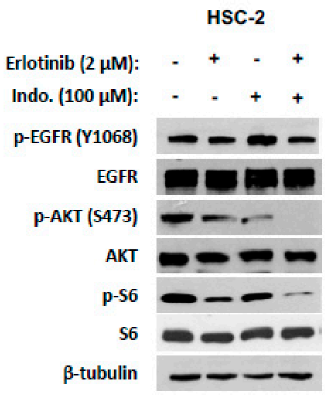

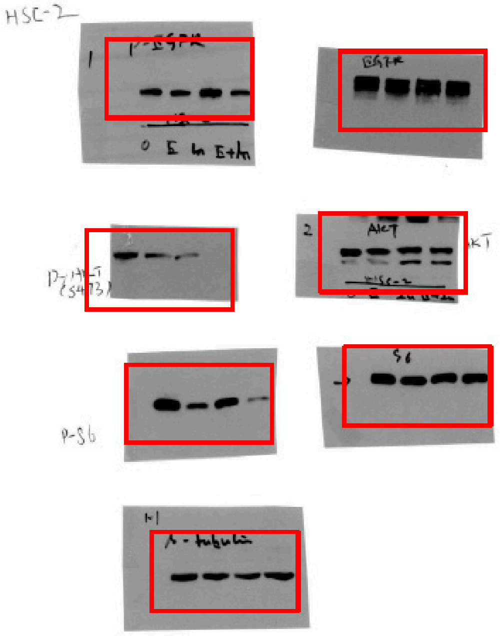

The red squares in the right image show the original ones in the left figures

Fig3. B

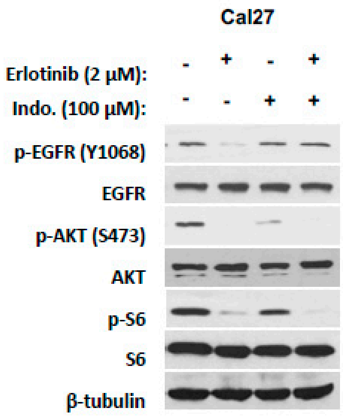

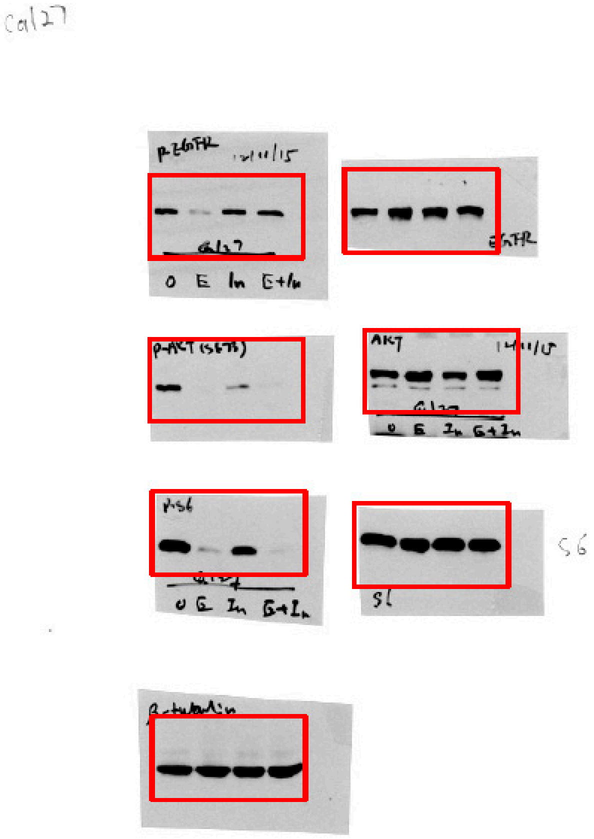

The red squares in the right image show the original ones in the left figures

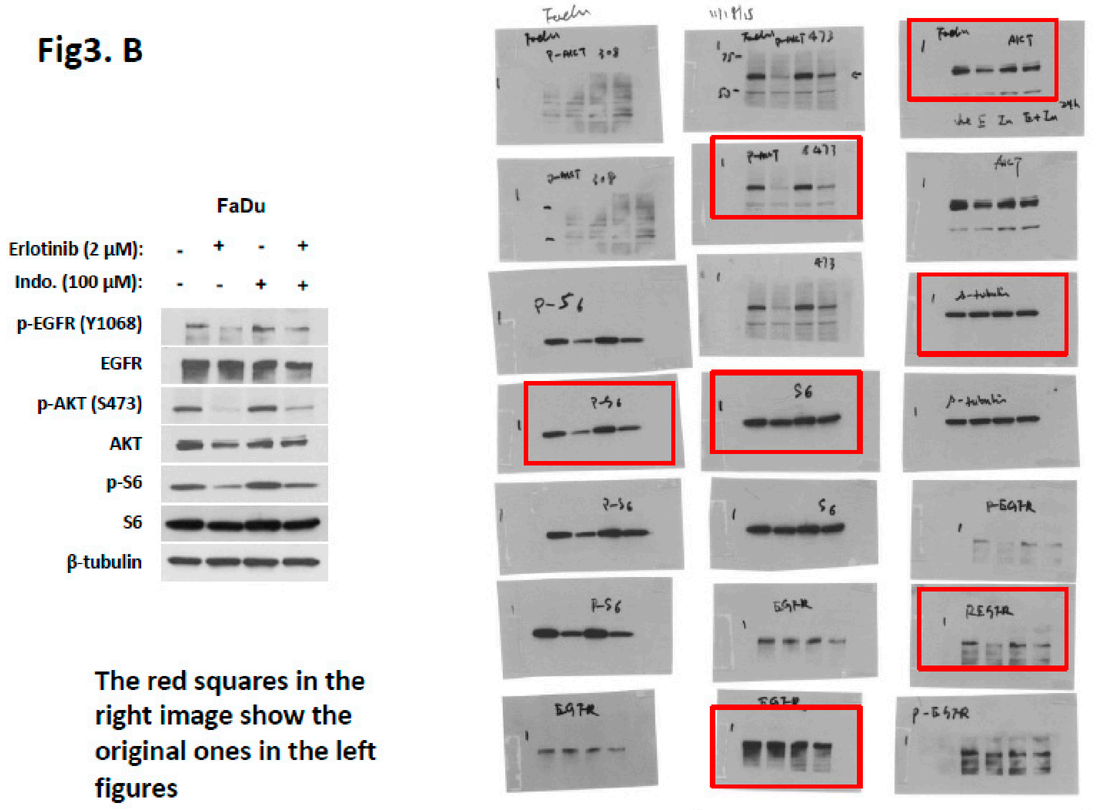

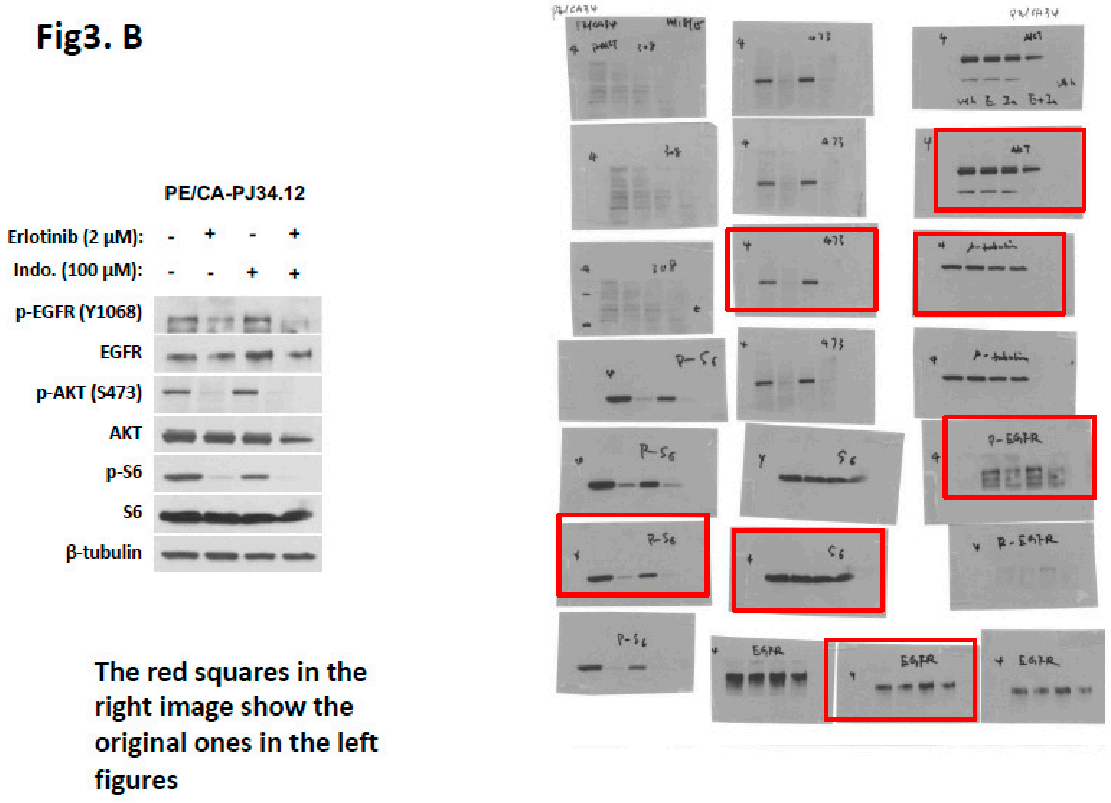

Figure S2. (**A**) The uncropped Western blots of Figure S1; (**B**) The uncropped Eestern blots of Figure 3b.
